# Seroprevalence of Infectious Bronchitis Virus Antibodies in Vaccinated Broilers from South-Western Romania (2018–2021): An ELISA-Based Survey

**DOI:** 10.3390/vetsci13050420

**Published:** 2026-04-25

**Authors:** Gabriel Orghici, Livia Stanga, Paula Nistor, Vlad Iorgoni, Marius Stelian Ilie, Diana Hoffman, Viorel Herman

**Affiliations:** 1 Department of Infectious Diseases and Preventive Medicine, Faculty of Veterinary Medicine, University of Life Sciences “King Mihai I” from Timisoara, 300645 Timisoara, Romania; gabriel.orghici@usvt.ro (G.O.); paula.nistor@usvt.ro (P.N.); vlad.iorgoni@usvt.ro (V.I.); viorel.herman@fmvt.ro (V.H.); 2 Discipline of Microbiology, Faculty of Medicine, “Victor Babes” University of Medicine and Pharmacy, Eftimie Murgu Square 2, 300041 Timisoara, Romania; 3 Discipline of Parasitology, Faculty of Veterinary Medicine, University of Life Sciences “King Mihai I” from Timisoara, 300645 Timisoara, Romaniadiana.hoffman@usvt.ro (D.H.); 4 Academy of Romanian Scientists (AOSR), Splaiul Independenței 54, 050094 Bucharest, Romania

**Keywords:** infectious bronchitis virus, IBV, broilers, seroprevalence, ELISA, antibodies, Romania, south-western Romania, poultry, vaccination monitoring

## Abstract

Infectious bronchitis virus (IBV) is an avian coronavirus associated with respiratory, renal, and reproductive disease in chickens, with important economic consequences in intensive poultry production. This study aimed to estimate the seroprevalence of anti-IBV antibodies in vaccinated broiler flocks reared in south-western Romania and to characterize its distribution across counties and study years. Between 2018 and 2021, a total of 2466 blood samples were collected from Ross 308 broilers aged 35–45 days originating from five commercial farms (one per county) located in Caraș-Severin, Dolj, Gorj, Hunedoara, and Vâlcea. Samples were obtained from 137 production halls/series. Sera were tested using a commercial indirect ELISA kit and classified according to the manufacturer’s criteria based on the sample-to-positive (S/P) ratio and the corresponding antibody titer threshold. Overall, 2115/2466 sera were positive (85.77%) and 351/2466 were negative (14.23%). Anti-IBV antibodies were detected in all halls (137/137, 100%), although negative sera were recorded in 87/137 halls (63%). Seroprevalence by county ranged from 78.67% (Vâlcea) to 89.24% (Hunedoara). Significant differences in the proportions of positive and negative sera were identified between several county pairs by Fisher’s exact test. These findings indicate widespread serological evidence of anti-IBV antibodies in vaccinated broiler flocks from south-western Romania, which may reflect vaccine-induced immunity and/or field exposure. The results support the need for continued serological monitoring, alongside targeted molecular investigations to differentiate vaccine strains from circulating field variants.

## Simple Summary

Infectious bronchitis virus (IBV) remains a major health and economic concern in commercial poultry production. Because broiler flocks are routinely vaccinated, serological monitoring is useful for assessing antibody responses at the flock level, although it cannot distinguish vaccine-derived antibodies from those associated with field exposure. In this study, we analyzed 2466 serum samples collected from vaccinated broilers aged 35–45 days reared in five counties of south-western Romania between 2018 and 2021. Anti-IBV antibodies were detected in 85.77% of samples, and all investigated production halls had at least one seropositive bird. However, 14.23% of birds were seronegative, and many halls included both positive and negative individuals, suggesting heterogeneous serological responses within flocks. These findings provide regional baseline data for vaccination monitoring and support the value of continued serological surveillance, complemented by molecular methods when field strain characterization is required.

## 1. Introduction

Avian infectious bronchitis (IB) is one of the most widespread and economically significant viral diseases affecting domestic chickens worldwide. Caused by infectious bronchitis virus (IBV), a member of the genus *Gammacoronavirus* within the family *Coronaviridae*, the disease is highly contagious and capable of infecting birds of all ages [1,2]. Infected flocks can experience respiratory distress, decreased weight gain, poor feed conversion, nephritis, reproductive disorders in layers, and, ultimately, significant production losses. The clinical manifestation of IB is influenced by factors such as viral strain, host age, concurrent infections, and environmental conditions [3,4]. Control of IB is complicated by the extensive genetic and antigenic variability in IBV, new variants continually emerge through mutations and recombination, challenging existing vaccine strategies and diagnostic approaches [1].

Vaccination remains the primary tool for IB control in commercial poultry operations. Live attenuated and inactivated vaccines are administered according to structured vaccination programs with the goal of inducing protective immunity before exposure to field strains [5,6]. However, the success of vaccination in broilers is dependent on several factors, including the match between vaccine and circulating strains, vaccine handling and administration, flock management, and the host’s immune competence [7]. Incomplete or uneven immune responses can leave subpopulations within flocks susceptible to infection, facilitating viral circulation and economic losses even under routine vaccination protocols [4,8].

Serological monitoring is widely adopted in poultry health programs to assess flock immunity following vaccination and to provide indirect evidence of viral exposure in field conditions. Enzyme-linked immunosorbent assays (ELISAs) are among the most commonly used serological tests due to their suitability for high-throughput screening and quantitative output, allowing for the estimation of antibody levels across large numbers of samples [8,9]. ELISA-based serology offers practical benefits for routine surveillance, enabling producers and veterinarians to monitor temporal trends in antibody prevalence and to compare responses across flocks and production cycles. However, ELISAs detect antibodies against antigenic determinants shared across IBV variants and vaccine strains, and they do not provide serotype or genotype resolution. As a result, serological data must be interpreted with caution, and elevated antibody titers often require follow-up with virus-specific molecular methods to distinguish vaccine-derived responses from natural infection [1,9]. In vaccinated broilers, ELISA-derived seroprevalence should, therefore, not be interpreted as direct evidence of field infection, because vaccine-induced and infection-induced antibodies cannot be distinguished by this approach alone. The poultry industry plays a significant role in the agricultural sector of many countries, including Romania, where broiler meat production constitutes a key component of national protein supply and economic activity. National livestock statistics indicate substantial poultry populations with fluctuations over time, reflecting dynamic production patterns and market demands [10,11]. Respiratory diseases with high transmissibility and persistent control challenges, such as IB, therefore, have practical implications for poultry health management and production efficiency. Despite its recognized importance, multi-year seroepidemiological information on IBV in Romanian broiler flocks remains limited, particularly for vaccinated broilers sampled close to slaughter age and compared across multiple counties within the same geographic region [12,13].

Seroprevalence studies conducted in other countries have demonstrated wide variability in IBV antibody prevalence in commercial poultry, influenced by vaccination practices, virus circulation, and local production systems [8,14]. Establishing baseline seroprevalence and understanding its distribution across time and space within a region provides a valuable epidemiological framework. Such knowledge facilitates interpretation of flock immunity status, benchmarking of vaccination outcomes, and the identification of potential areas for enhanced disease surveillance or adjustment of immunization strategies [12,15].

In this context, the present study aimed to quantify the seroprevalence of anti-IBV antibodies in vaccinated broiler flocks sampled at 35–45 days of age from south-western Romania over a four-year period (2018–2021), as well as to characterize the distribution of serological status across counties, study years, and production halls using ELISA-based testing. By providing multi-year regional data from vaccinated broilers at the end of the production cycle, this study was intended to contribute baseline information for serological surveillance and vaccination monitoring in Romanian poultry production.

## 2. Materials and Methods

### 2.1. Study Area and Sampling Design

**Figure 1:**
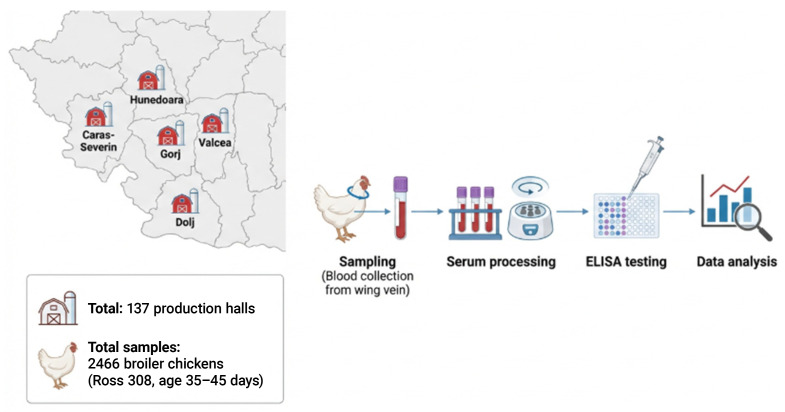
Schematic representation of the study design and workflow.

A cross-sectional serological survey was conducted between 2018 and 2021 in the south-western region of Romania. Serum samples were collected from commercial broiler farms located in five counties: Caraș-Severin (CS), Dolj (DJ), Gorj (GJ), Hunedoara (HD), and Vâlcea (VL). One commercial broiler farm was included from each county. Farm inclusion was opportunity-based, depending on farm accessibility, sample availability, and owner willingness to participate; therefore, the selected farms should not be considered fully representative of all broiler farms in each county. All farms included in the survey had a documented history of respiratory problems, defined as the occurrence of clinical respiratory signs and/or recorded outbreaks in previous production cycles. At the time of sampling, some flocks also exhibited mild to moderate respiratory signs, although sampling was not restricted to actively diseased flocks. Sampling was performed in 137 broiler production units (halls/series) across the four-year study period. In this study, a “production hall” refers to a poultry house containing one broiler flock/production series managed as a distinct production and epidemiological unit. The overall study design, including sampling strategy and laboratory workflow, is illustrated in Figure 1.

A cross-sectional serological survey was conducted between 2018 and 2021 in five counties from south-western Romania. Blood samples were collected from broiler chickens (Ross 308, 35–45 days of age) across 137 production halls. Serum samples were analyzed by ELISA for anti-IBV antibodies, followed by statistical evaluation of seroprevalence.

### 2.2. Animals

The study population consisted of Ross 308 broiler chickens aged 35–45 days. Information regarding the hatchery source of broiler chicks was not consistently available for all farms and production cycles and was, therefore, not included in the analysis. All farms included in the survey had a history of respiratory problems, and broilers were routinely vaccinated against the main respiratory pathogens, including IBV, according to farm-specific vaccination programs. Farm inclusion was opportunity-based, depending on farm accessibility, sample availability, and owner willingness to participate. In addition, all included farms had a documented history of respiratory problems, which represented an inclusion criterion for sampling. This selection approach may have increased the likelihood of detecting higher seroprevalence levels compared to the general broiler population and should be considered when interpreting the results. Broilers were vaccinated against IBV according to routine farm-specific programs, typically involving live attenuated vaccines administered during the first weeks of life. In most cases, vaccination was performed once or twice, commonly at day 1 and/or during the first 2–3 weeks. However, detailed information regarding vaccine strains, administration routes, and exact schedules was not consistently available. Blood samples were collected at 35–45 days of age, corresponding to approximately 2–4 weeks post-vaccination, depending on the farm protocol. Detailed information on vaccine type, strain, route of administration, and exact vaccination schedule was not consistently available for all sampled production cycles and, therefore, could not be standardized in the present analysis.

### 2.3. Sample Collection and Serum Processing

Blood sampling was performed by collecting 1–2 mL of blood from the wing vein (brachial vein) using sterile disposable syringes [16]. Samples were transferred into tubes without anticoagulant and kept in a vertical position at room temperature until clot formation. Serum was obtained by decantation and transferred into sterile Eppendorf tubes [17]. Serum samples were transported to the laboratory under refrigerated conditions (cold chain maintained) and centrifuged at 3000 rpm for 5 min. After centrifugation, sera were stored at −20 °C until analysis [18,19].

### 2.4. Serological Testing (ELISA)

All serum samples were tested for the presence of anti-IBV antibodies using a commercial indirect ELISA kit for chickens (Infectious Bronchitis Virus Antibody Test Kit, catalogue no. CK119 IBV, BioChek, Reeuwijk, The Netherlands), according to the manufacturer’s instructions [20]. The assay is designed for the detection of antibodies against known infectious bronchitis virus (IBV) strains in chicken serum.

Briefly, serum samples were diluted to a final working dilution of 1:500 in the provided dilution buffer. For assay setup, 100 µL of negative control sera was added to wells A1 and B1, and 100 µL of positive control sera was added to wells C1 and D1 of the antigen-coated microtiter plate. The remaining wells received 100 µL of diluted serum samples. Plates were incubated for 30 min at room temperature, washed four times with wash buffer, and then incubated with conjugate reagent for an additional 30 min at room temperature. After a second washing step, substrate solution was added and the plates were incubated for 15 min at room temperature. The reaction was stopped by adding 100 µL of stop solution, and optical density (OD) was measured at 405 nm within 15 min after stopping the reaction [20].

The results were interpreted by calculating the sample-to-positive (S/P) ratio according to the manufacturer’s instructions, using the formula S/P = (ODsample − ODnegative control)/(ODpositive control − ODnegative control). Each ELISA plate included kit-provided positive and negative control sera. The assay was considered valid when the optical density (OD) values of controls met the acceptance criteria specified by the manufacturer, including a sufficient difference between positive and negative controls and appropriate S/P ratio values. Based on the BioChek kit criteria, samples with an S/P ratio ≥ 0.2 were considered positive, corresponding to an antibody titer ≥ 396, while samples with an S/P ratio < 0.2 were considered negative [20,21]. Samples were classified as positive or negative for anti-IBV antibodies using the kit-specific threshold.

Positive and negative controls supplied with the kit were included on each plate, and assay validity was assessed according to the manufacturer’s acceptance criteria. Because this indirect ELISA detects anti-IBV antibodies without strain-level discrimination, the results were interpreted as evidence of serological response and not as direct proof of infection with a specific field strain.

### 2.5. Data Management and Statistical Analysis

For each serum sample, the ELISA status (positive/negative) and antibody titer were recorded. Data were grouped by county and by year of sampling.

Descriptive statistics were used to summarize the distribution of ELISA results. The proportions of positive and negative samples were calculated for the total dataset and for each county and year, together with 95% confidence intervals (95% CIs). Differences in proportions between counties were assessed using Fisher’s exact test. Because multiple pairwise county comparisons were performed, *p*-values were interpreted with caution and, where applicable, adjusted for multiple testing. Year-specific seroprevalence values were analyzed descriptively. Statistical analysis was performed using GraphPad Prism 9.0 Software (GraphPad Software, San Diego, CA, USA), GraphPad QuickCalcs (GraphPad Software San Diego, CA, USA), and Microsoft Office Excel 2016 (Microsoft Corporation, Redmond, WA, USA) [22]. Because samples were clustered within production halls and farms, the inferential analysis should be interpreted as exploratory rather than as a fully independent-sample model.

## 3. Results

### 3.1. Overall Serological Status of the Study Population

The data collected from broiler chickens (Ross 308, 35–45 days of age) originating from 137 production halls were tested by ELISA for anti-IBV antibodies. Anti-IBV antibodies were detected in 2115/2466 sera, resulting in an overall seroprevalence of 85.77% (95% CI: 84.39–87.15%). A total of 351/2466 sera were negative, representing 14.23% of the tested samples.

At the hall level, at least one positive serum sample was identified in all production halls included in the study (137/137; 100%). However, negative sera were also recorded in 87/137 halls (63%), indicating that a substantial proportion of halls contained both positive and negative individuals and, therefore, showed within-hall heterogeneity in serological status.

### 3.2. Distribution of Samples by County and Study Year

The number of serum samples collected varied across counties and years. Across the four-year period, the highest number of sera originated from Gorj County (746/2466), followed by Caraș-Severin (478/2466), Hunedoara (437/2466), Dolj (430/2466), and Vâlcea (375/2466). The distribution of samples by year was 517 sera in 2018, 747 in 2019, 606 in 2020, and 596 in 2021.

At the hall level, a total of 137 halls were surveyed, including 30 halls in 2018, 43 in 2019, 33 in 2020, and 31 in 2021. Gorj County contributed the highest number of halls (42), while Hunedoara contributed the lowest (22). In this study, a “production hall” refers to a poultry house containing a single broiler flock managed as a distinct epidemiological unit.

### 3.3. Seroprevalence by County

**Figure 2:**
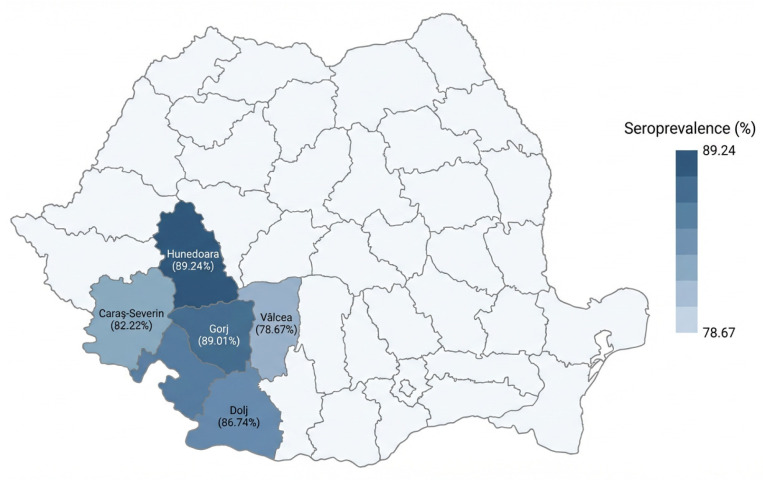
Geographic distribution of anti-IBV antibody seroprevalence in broiler flocks from five counties in south-western Romania. Seroprevalence values ranged from 78.67% in Vâlcea to 89.24% in Hunedoara.

**Figure 3:**
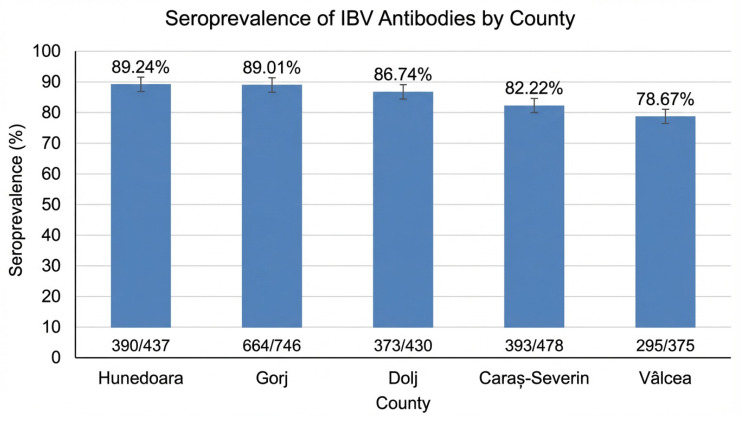
County-level seroprevalence of anti-IBV antibodies in broiler chickens, shown with 95% confidence intervals. Seropositive samples accounted for 390/437 in Hunedoara, 664/746 in Gorj, 373/430 in Dolj, 393/478 in Caraș-Severin, and 295/375 in Vâlcea.

Seroprevalence differed across the five counties included in the survey, indicating moderate geographic variability in serological status among the investigated farms. The highest proportion of positive sera was recorded in Hunedoara County (89.24%, 390/437), followed by Gorj (89.01%, 664/746), Dolj (86.74%, 373/430), Caraș-Severin (82.22%, 393/478), and Vâlcea (78.67%, 295/375). The highest proportion of negative sera was observed in Vâlcea County (21.33%, 80/375), while the lowest was recorded in Hunedoara County (10.76%, 47/437). The geographical distribution of seroprevalence across the five counties is presented in Figure 2. Differences in seroprevalence between counties are further illustrated in Figure 3.

The observed differences in seroprevalence between counties may reflect variations in farm management, vaccination practices, or local epidemiological pressure. Higher seroprevalence values could indicate more consistent vaccine-induced immunity or increased exposure to IBV, whereas lower values may suggest less uniform seroconversion within flocks.

### 3.4. Seroprevalence by Study Year

**Figure 4:**
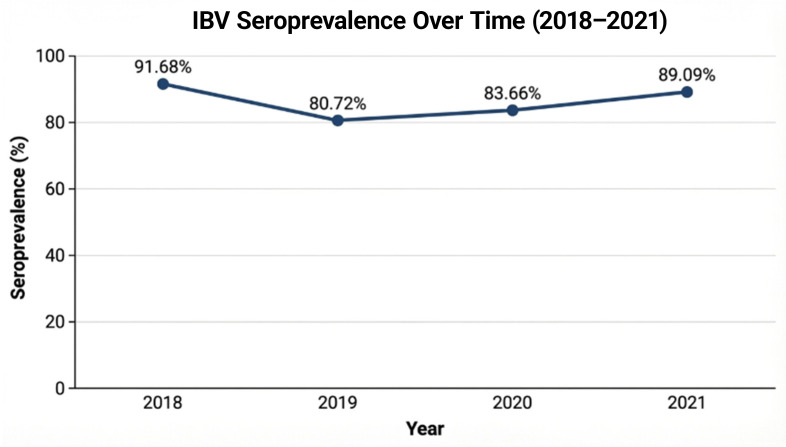
Temporal variation in anti-IBV antibody seroprevalence in broiler flocks from 2018 to 2021. The highest seroprevalence was recorded in 2018 (91.68%), while the lowest occurred in 2019 (80.72%).

Across the four study years, anti-IBV antibodies were detected in the majority of tested sera. In 2018, 474/517 sera were positive (91.68%) and 43/517 were negative (8.32%). In 2019, 603/747 sera were positive (80.72%) and 144/747 were negative (19.28%). In 2020, 507/606 sera were positive (83.66%) and 99/606 were negative (16.34%). In 2021, 531/596 sera were positive (89.09%) and 65/596 were negative (10.91%). Temporal variation in seroprevalence across the study period is shown in Figure 4; these year-to-year differences were described descriptively.

Year-to-year variation in seroprevalence may reflect temporal differences in vaccination practices, farm management, or infection pressure, although these observations are descriptive and should be interpreted with caution.

### 3.5. Statistical Comparison Between Counties

Statistical analysis using Fisher’s exact test indicated significant differences in the proportions of positive and negative sera between several county pairs. Significant differences were identified for Caraș-Severin vs. Gorj (*p* = 0.0009), Caraș-Severin vs. Hunedoara (*p* = 0.0029), Dolj vs. Vâlcea (*p* = 0.0026), Gorj vs. Vâlcea (*p* = 0.0001), and Hunedoara vs. Vâlcea (*p* = 0.0001). No significant differences were detected between the other county comparisons.

### 3.6. Summary of Hall-Level Results

At the hall level, all 137 investigated production halls had at least one seropositive sample, whereas 87 halls (63%) also included seronegative birds, indicating within-hall heterogeneity in serological status. Negative sera were identified in halls from all five counties included in the study. At the production hall level, the proportion of seropositive birds showed considerable variability. The median within-hall seroprevalence was 88.4%, with values ranging from 52.0% to 100% across the 137 halls. A subset of halls exhibited complete seropositivity (100% positive samples), whereas others showed mixed serological status with varying proportions of seronegative individuals.

**Figure 5:**
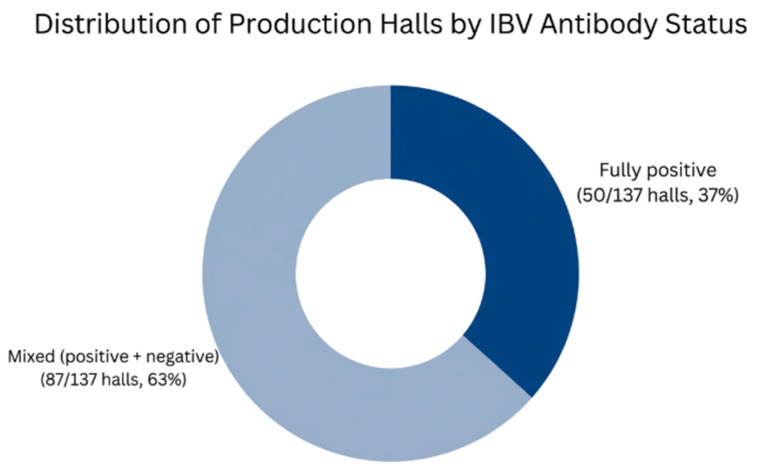
Distribution of production halls according to serological status. All investigated halls (137/137) contained at least one seropositive sample, while 63% (87/137) also included seronegative individuals, indicating heterogeneous seroconversion within flocks.

Overall, the results demonstrate widespread detection of anti-IBV antibodies in broiler flocks from south-western Romania during 2018–2021, with variability in seroprevalence between counties and across study years. The distribution of positive and mixed-status production halls is summarized in Figure 5.

The within-hall heterogeneity observed in a substantial proportion of production units suggests uneven immune responses at the flock level, which may have implications for viral persistence and transmission dynamics within broiler populations.

## 4. Discussion

This multi-year serological survey provides evidence of widespread anti-IBV antibody detection in vaccinated broiler flocks from south-western Romania. Overall, 85.77% of sera (2115/2466) were ELISA-positive, and antibodies were detected in all investigated halls (137/137). These findings indicate widespread serological evidence of anti-IBV antibodies in vaccinated broiler flocks from the investigated region, likely reflecting vaccine-induced immunity and possibly field exposure, while also supporting the continued relevance of IBV in intensive broiler production systems [23]. The overall seroprevalence observed in this study is comparable with reports from other countries where IBV vaccination is routinely implemented. Studies conducted in different geographic regions have frequently documented high proportions of seropositive birds in commercial broiler populations, often exceeding 70–80%, and in some settings approaching 100% [1,24]. Such results are generally interpreted as compatible with vaccination-induced serological response and, in some settings, possible field exposure, particularly when sampling is performed at later broiler ages (e.g., 35–45 days), when the time window for seroconversion is sufficient [4,25]. The high seroprevalence reported here is, therefore, consistent with expectations for intensively reared broilers under vaccination programs and aligns with the epidemiological behavior of IBV as a highly transmissible virus capable of persisting in poultry-dense production areas [12,26,27].

The present study provides multi-year, region-specific data on IBV seroprevalence in vaccinated broiler flocks from south-western Romania, a context for which longitudinal seroepidemiological information remains limited. By integrating data across multiple counties and production cycles, this dataset contributes baseline values for vaccination monitoring and supports regional epidemiological mapping of IBV in commercial poultry systems.

Despite the overall high proportion of positive sera, a relevant finding of this study was the detection of negative samples in 14.23% of tested sera (351/2466) and the presence of negative sera in 63% of halls (87/137). This observation suggests that, although exposure and/or vaccination response is widespread, seroconversion may be incomplete or uneven within many broiler flocks. In broiler production, variability in antibody status can result from differences in vaccine administration quality (e.g., spray distribution, drinking water delivery), timing of vaccination relative to maternal antibody decline, or heterogeneity in immune response among individuals [7,28]. In addition, the presence of seronegative birds at 35–45 days of age may reflect suboptimal vaccine uptake or failure to mount detectable antibody levels. From a practical standpoint, this pattern is important because susceptible subgroups within flocks may enable viral circulation and contribute to ongoing respiratory problems, even when the majority of birds are seropositive [29]. In partially immune flocks, heterogeneous serological response may also reflect uneven vaccine uptake, individual variability in immune response, or differences in the timing of seroconversion. Marked differences in seroprevalence were also observed between counties, with values ranging from 78.67% (Vâlcea) to 89.24% (Hunedoara). Statistical comparisons identified significant differences in the proportions of positive and negative sera between several county pairs. Although the present study design does not allow causal attribution, these differences may be related to unmeasured variation in farm management, biosecurity, vaccination practices, chick sourcing, or local epidemiological conditions; however, these factors were not directly assessed in the present study and no causal inference can be made. In particular, a lower seroprevalence could indicate weaker vaccine take or reduced exposure, while higher seroprevalence may reflect more consistent vaccine-induced immunity and/or more frequent field challenges. Importantly, because ELISAs detect IBV antibodies broadly and do not distinguish vaccine-induced from infection-induced antibodies, the observed differences should be interpreted as differences in serological status rather than direct evidence of differing field strain circulation [9,30,31].

Variation across study years was also evident. The highest proportion of seropositive samples was recorded in 2018 (91.68%), while the lowest was recorded in 2019 (80.72%). These descriptive year-to-year differences may reflect temporal variation in vaccination practices, farm management, or epidemiological pressure; however, no formal causal interpretation should be made from the present dataset [5,32]. The year-to-year variation highlights the importance of continuous monitoring rather than relying on single-year datasets when evaluating regional IBV epidemiology and the effectiveness of prevention programs [33]. Although temporal variation in seroprevalence was observed, no direct correlation with changes in vaccination protocols could be established, as detailed year-specific vaccination data were not available. The study period partially overlapped with the COVID-19 pandemic; however, no consistent information was available regarding potential impacts on vaccine availability, farm management, or data collection. Therefore, no specific conclusions could be drawn regarding the influence of pandemic-related factors on the results.

A strength of the present study is the relatively large sample size (2466 sera) collected over four consecutive years, allowing for the robust estimation of seroprevalence and temporal patterns. Additionally, sampling was performed at 35–45 days of age, which is a relevant time window for broilers because it reflects the immunity status close to slaughter age and captures both vaccine-induced seroconversion and potential field exposure occurring during the production cycle. This age-targeted approach provides practical value for broiler health management.

However, several limitations must be considered when interpreting these findings. First, the study relied exclusively on ELISA-based serology, which is appropriate for large-scale monitoring but cannot differentiate antibodies induced by vaccination from those induced by natural infection, nor can it identify circulating IBV genotypes [9,30]. Second, only one farm per county was included, and farm selection was opportunity-based; therefore, county-level findings should not be considered fully representative of all broiler farms in each county. Third, all included farms had a history of respiratory problems, which may have increased the likelihood of detecting high seropositivity and may limit generalizability to the wider broiler population. Fourth, detailed vaccination records and farm-level management variables were not consistently available for comparative analysis. The lack of information on hatchery origin represents a limitation, as differences in hatchery vaccination programs, maternal antibody levels, and early-life exposure could have influenced the observed serological patterns. An important limitation of ELISA-based serology is that it reflects systemic humoral immunity and does not capture local mucosal immune responses or cell-mediated immunity, both of which play a critical role in protection against IBV. In particular, mucosal IgA responses in the upper respiratory tract and T-cell-mediated immunity may confer protection even in birds with low or undetectable serum antibody levels. Conversely, high serum antibody titers do not necessarily prevent infection or viral replication in the respiratory tract, especially in the case of heterologous or antigenically divergent IBV strains. Finally, samples were clustered within production halls and farms, meaning that observations were not fully independent from an epidemiological standpoint.

Future research should combine serology with the molecular detection and characterization of IBV (e.g., RT-PCR targeting the S1 gene and sequencing) from respiratory samples collected during clinical outbreaks or from flocks showing serological patterns suggestive of stronger field challenge [1,14,34]. Such investigations would enable identification of circulating genotypes and evaluation of vaccine matching, which is essential given the rapid evolution and diversity of IBV [26,32]. In addition, integrating farm-level data (vaccination schedule, administration route, biosecurity measures, and performance indicators) would allow more detailed risk factor analysis and provide actionable recommendations for optimizing IBV control programs in Romania.

## 5. Conclusions

This study provides multi-year regional baseline data on the serological status of vaccinated broiler flocks from south-western Romania at 35–45 days of age. Anti-IBV antibodies were widely detected across the investigated farms and production halls, indicating that serological response to IBV is common under routine field conditions. At the same time, the presence of seronegative birds and mixed-status halls highlights heterogeneity in flock-level serological responses and supports the need for the continued monitoring of vaccination outcomes. These findings are relevant for regional IBV surveillance and provide a useful basis for future studies integrating vaccination records, farm-level risk factors, and the molecular characterization of circulating strains.

## Data Availability

The data presented in this study are available within the article. Further inquiries can be directed to the corresponding author.

## Abbreviations

The following abbreviations are used in this manuscript:

IB	Infectious bronchitis
IBV	Infectious bronchitis virus
ELISA	Enzyme-linked immunosorbent assay
RT-PCR	Reverse transcription polymerase chain reaction
OD	Optical density
S/P	Sample-to-positive ratio
CS	Caraș-Severin
DJ	Dolj
GJ	Gorj
HD	Hunedoara
VL	Vâlcea

## References

[1] Lin S.Y., Chen H.W. (2017). Infectious bronchitis virus variants: Molecular analysis and pathogenicity investigation. Int. J. Mol. Sci..

[2] Muradrasoli S., Bálint A., Wahlgren J., Waldenström J., Belák S., Blomberg J., Olsen B. (2010). Prevalence and phylogeny of coronaviruses in wild birds from the Bering Strait area (Beringia). PLoS ONE.

[3] Amarasinghe A., Abdul-Cader M.S., Nazir S., De Silva Senapathi U., van der Meer F., Cork S.C., Gomis S., Abdul-Careem M.F. (2017). Infectious bronchitis coronavirus establishes productive infection in avian macrophages interfering with selected antimicrobial functions. PLoS ONE.

[4] Lopes P.D., Okino C.H., Fernando F.S., Pavani C., Casagrande V.M., Lopez R.F.V., Montassier M.F.S., Montassier H.J. (2018). Inactivated infectious bronchitis virus vaccine encapsulated in chitosan nanoparticles induces mucosal immune responses and effective protection against challenge. Vaccine.

[5] Franzo G., Tucciarone C.M., Blanco A., Nofrarías M., Biarnés M., Cortey M., Majó N., Catelli E., Cecchinato M. (2016). Effect of different vaccination strategies on IBV QX population dynamics and clinical outbreaks. Vaccine.

[6] Laconi A., Weerts E.A.W.S., Bloodgood J.C.G., Deniz Marrero J.P., Berends A.J., Cocciolo G., de Wit J.J., Verheije M.H. (2020). Attenuated live infectious bronchitis virus QX vaccine disseminates slowly to target organs distant from the site of inoculation. Vaccine.

[7] Al-Rasheed M., Ball C., Parthiban S., Ganapathy K. (2023). Evaluation of protection and immunity induced by infectious bronchitis vaccines administered by oculonasal, spray or gel routes in commercial broiler chicks. Vaccine.

[8] van Ginkel F.W., Padgett J., Martinez-Romero G., Miller M.S., Joiner K.S., Gulley S.L. (2015). Age-dependent immune responses and immune protection after avian coronavirus vaccination. Vaccine.

[9] Gu K., Song Z., Ma P., Liao Z., Yang M., Zhou C., Li C., Zhao Y., Li H., Yang X. (2022). A novel nanobody-horseradish peroxidase fusion based competitive ELISA to rapidly detect avian coronavirus infectious bronchitis virus antibody in chicken serum. Int. J. Mol. Sci..

[10] National Institute of Statistics (Romania) (2026). TEMPO-Online Database, AGR201C: Poultry.

[11] Eurostat Poultry Statistics. European Commission, Statistics Explained. https://ec.europa.eu/eurostat/statistics-explained/index.php?title=Poultry_statistics.

[12] Franzo G., Massi P., Tucciarone C.M., Barbieri I., Tosi G., Fiorentini L., Ciccozzi M., Lavazza A., Cecchinato M., Moreno A. (2017). Phylodynamic reconstruction of infectious bronchitis virus QX genotype reveals different population dynamics and spreading patterns. PLoS ONE.

[13] Orghici G.I., Ilie M.S., Iorgoni V.I., Dreghiciu I.C., Hoffman D., Herman V.I. Serological assessment of IBV in broilers from Dolj County. Proceedings of the Multidisciplinary Conference on Sustainable Development.

[14] Jiang Y., Cheng X., Zhao X., Yu Y., Gao M., Zhou S. (2020). Recombinant infectious bronchitis coronavirus H120 with the spike protein S1 gene of the nephropathogenic IBYZ strain remains attenuated but induces protective immunity. Vaccine.

[15] Li J., Helal Z.H., Karch C.P., Mishra N., Girshick T., Garmendia A., Burkhard P., Khan M.I. (2018). A self-adjuvanted nanoparticle based vaccine against infectious bronchitis virus. PLoS ONE.

[16] Morishita T.Y. (2019). Poultry Blood Collection.

[17] Hy-Line International Proper Collection and Handling of Diagnostic Samples—Part One: Serology and Blood Collection.

[18] Cadamuro J., Mrazek C., Leichtle A.B., Kipman U., Felder T.K., Wiedemann H., Oberkofler H., Fiedler G.M., Haschke-Becher E. (2018). Influence of centrifugation conditions on routine clinical chemistry analytes. Biochem. Med..

[19] World Health Organization (2012). Surveillance Guidelines for Measles, Rubella and Congenital Rubella Syndrome in the WHO European Region.

[20] BioChek (2026). Infectious Bronchitis Virus Antibody Test Kit, CK119 IBV.

[21] Crowther J.R. (2009). The ELISA Guidebook.

[22] Kim H.Y. (2017). Statistical notes for clinical researchers: Chi-squared test and Fisher’s exact test. Restor. Dent. Endod..

[23] van Beurden S.J., Berends A.J., Krämer-Kühl A., Spekreijse D., Chenard G., Philipp H.C., Mundt E., Rottier P.J.M., Verheije M.H. (2018). Recombinant live attenuated avian coronavirus vaccines protect against infectious bronchitis. Vaccine.

[24] Whitehead A.B.R., Butcher G.D., Walden H.S., Duque V., Cruz M., Hernandez J.A. (2018). Burden of exposure to infectious pathogens in broiler chickens. PLoS ONE.

[25] Lublin A., Katz C., Gruzdev N., Yadid I., Bloch I., Farnoushi Y., Simanov L., Berkowitz A., Elyahu D., Pitcovski J. (2022). Protection against avian coronavirus conferred by oral vaccination. Vaccine.

[26] Franzo G., Cecchinato M., Tosi G., Fiorentini L., Faccin F., Tucciarone C.M., Trogu T., Barbieri I., Massi P., Moreno A. (2018). GI-16 lineage history and epidemiology. PLoS ONE.

[27] Bilotti K., Keep S., Sikkema A.P., Pryor J.M., Kirk J., Foldes K., Doyle N., Wu G., Freimanis G., Dowgier G. (2024). One-pot golden gate assembly of an avian infectious bronchitis virus reverse genetics system. PLoS ONE.

[28] Ganapathy K., Bufton A., Pearson A., Lemiere S., Jones R.C. (2010). Vaccination of broiler chicks against avian metapneumovirus infection. Vaccine.

[29] Le Gros F.X., Dancer A., Giacomini C., Pizzoni L., Bublot M., Graziani M., Prandini F. (2009). Field efficacy trial of a novel vector vaccine for broilers. Vaccine.

[30] Liu I.L., Lin Y.C., Lin Y.C., Jian C.Z., Cheng I.C., Chen H.W. (2019). A novel immunochromatographic strip for antigen detection of IBV. Int. J. Mol. Sci..

[31] Legnardi M., Tucciarone C.M., Franzo G., Cecchinato M. (2020). Infectious Bronchitis Virus Evolution, Diagnosis and Control. Vet. Sci..

[32] Jackwood M.W., Lee D.H. (2017). Evolutionary trajectories of vaccine-controlled and non-controlled IBV. PLoS ONE.

[33] Ingelbeen B., van Kleef E., Mbala P., Danis K., Macicame I., Hens N., Cleynen E., van der Sande M.A.B. (2025). Embedding risk monitoring in infectious disease surveillance. BMJ Glob. Health.

[34] Liu S., Zhang X., Gong L., Yan B., Li C., Han Z., Shao Y., Li H., Kong X. (2009). Altered pathogenicity and immunogenicity of IBV strain after serial passage. Vaccine.

